# The Effects of GLCM parameters on LAI estimation using texture values from Quickbird Satellite Imagery

**DOI:** 10.1038/s41598-017-07951-w

**Published:** 2017-08-04

**Authors:** Jingjing Zhou, Rui Yan Guo, Mengtian Sun, Tajiguli Tu Di, Shan Wang, Jiangyuan Zhai, Zhong Zhao

**Affiliations:** 1 College of Horticulture & Forestry Sciences/Hubei Engineering Technology Research Center for Forestry Information, Huazhong Agriculture University, Wuhan, Hubei 430070 P.R. China; 2 College of Forestry/Shaanxi comprehensive key laboratory of forestry, Northwest A&F University, Yangling, Shaanxi 712100 P.R. China

## Abstract

When the leaf area index (LAI) of a forest reaches 3, the problem of spectrum saturation becomes the main limitation to improving the accuracy of the LAI estimate. A sensitivity analysis of the Grey Level Co-occurrence Matrix (GLCM) parameters which can be applied to satellite image processing and analysis showed that the most important parameters included orientation, displacement and moving window size. We calculated the values of Angular Second Moment (ASM), Entropy (ENT), Correlation (COR), Contrast (CON), Dissimilarity (DIS) and Homogeneity (HOM) from Quickbird panchromatic imagery using a GLCM method. Four orientations, seven displacements and seven window sizes were considered. An orientation of 90° was best for estimating the LAI of black locust forest, regardless of moving window size, displacement and texture parameters. Displacements of 3 pixels appeared to be best. The orientation and window size had only a little influence on these settings. The highest adjusted r^2^ values were obtained using a 3 × 3 moving window size for ASM and ENT. The tendency of CON, COR, DIS and HOM to vary with window size was significantly affected by orientation. This study can help with parameter selection when texture features from high resolution imagery are used to estimate broad-leaved forest structure information.

## Introduction

Leaf area index (LAI) is an important input variable in forest ecosystem modelling as it is a factor in predicting productivity and assessing forest health over large areas^1^. Remote sensing technologies have become increasingly important in large-scale ecological studies because of their low cost and ability to provide large amounts of relevant information quickly. Passive optical remote sensing is the most widely used method for obtaining data for LAI estimation^2, 3^ although there are many studies that focus on LAI estimation using passive airborne laser scanners (Lidar)^4, 5^. Lidar does not saturate at high values (LAI > 3) and can better separate the understory which includes grasses, herbs and shrubs etc., distributed below the forest canopy. However, due to the high cost of the method, there are limited archives of Lidar images available for analyzing the change in vegetation structure over time^6^. Currently, the LAI distribution at a landscape scale or a regional scale of forest can be estimated effectively using passive optical remote sensing techniques, especially high resolution satellite remote sensing.

Spectral information has been widely used to analyze large areas of forest. LAI data can be obtained by analysing optical data using regression models based on spectral vegetation indices (SVIs). However, SVIs become saturated when LAI values are larger than 3^7, 8^. This phenomenon is a serious problem when analyzing forest environments exhibiting large heterogeneity with complex vertical and horizontal structures. This is one of the principal limitations to the improvement of LAI estimations of forest canopies. Many studies have demonstrated the potential of high resolution satellite remote sensing sensors (such as IKONOS and QuickBird) for estimating and mapping forest LAI spatially. Texture features, which are frequently used pieces of spatial information and derived from these high-resolution images, have proved to be effective for significantly increasing the accuracy of forest LAI estimation^3, 7, 9, 10^. Texture analysis involves the measurement of heterogeneity in the tonal values of pixels within a defined area of an image^6^ and can be used to identify objects or regions of interest^11^. Song & Dickinson^10^ demonstrated that image textural information was more useful for estimating LAI than two spectral vegetation indices. Zhou *et al*.^3^ confirmed that a combination of texture and SVIs can yield r^2^ values of 0.84 when they used Quickbird imagery to estimate the LAI of a black locust plantation. Pu & Cheng^9^ showed that texture-based features from Worldview-2 data are more useful than spectrum-based features and a combination of the two could lead to even higher accuracy of mapping forest LAIs than either one separately. The texture features in high resolution data provide better accuracy than using relatively low resolution data. These studies demonstrated that the accuracy of estimated forest LAI based on remote-sensing data could be significantly increased by considering textural information. Therefore, textural information derived from high resolution satellite imagery has been shown to be unique and can be very useful in estimating and mapping forest LAI.

Texture is a complex parameter and texture values measured with the GLCM method are highly sensitive to moving window size, orientation, displacement and physiographic conditions^3, 12–17^. The sensitivity of these texture parameters in relation to LAI estimation using GLCM has not been thoroughly studied^16^. How to set the value of the moving window size, orientation and displacement when extracting GLCM texture features is still confusing and literature about setting parameters of GLCM is relatively rare. For example, there is contradictory advice as to whether a large or small moving window size should be used. Some studies have shown that image texture measures calculated using a small window size from high resolution imagery were most strongly associated with vegetation structure as observed on the ground^3, 6^. Others considered that a small moving window size contributes to the sparsity and instability of the GLCM^18^. Coburn & Roberts^13^ showed that texture features cannot be described clearly by only using one moving window size. A small window size should be chosen when the type of land under investigation is homogeneous, as opposed to a large window size. Puissant *et al*.^16^ determined the best window size for land classification by comparing the variable coefficient of texture parameters extracted using different window sizes.

Displacement is another important factor influencing the value of GLCM parameters. Large pixel displacement leads to low comparability. The probability of occurrence of particular grey levels along the diagonal of the GLCM is small. However, there have been few studies that have investigated the effects of displacement on the texture features, or its potential to estimate LAI. Kayitakire *et al*.^15^ considered that the displacement and moving window size were the most sensitive input variables when they estimated forest structure parameters. However, in their research, the influence of displacement on texture features was not studied. The orientation used for the GLCM was also generally ignored, with the average value of texture from four orientations usually applied. The influence of orientation on texture features was not studied in detail. In the study by Kayitakire *et al*.^15^, orientation had little effect on the accuracy of forest parameter estimation. However, Clausi^19^ suggested that each orientation should be used to calculate texture value. Thus, the determination of orientation needs further analysis^13^.

In conclusion, the sensitivity of these GLCM parameters to the texture features has been studied to help choose the appropriate parameter values for estimating forest variables. Only rarely have these parameters been optimised. Instead, trial and error has been used with the user accepting the best possible option from those tried intuitively. There is a lack of deep understanding of how texture features work, and only locally-based studies have been found in the literature. It is therefore important to carry out a study into how to set the parameters for GLCM features. This work could contribute to the building of a deeper knowledge of the topic. Therefore, the main objective of this study was to test the influence of GLCM parameters on the LAI estimation of black locust plantations in mountain areas of the Loess Plateau in China by calculating texture features from a Quickbird panchromatic image. The GLCM parameters examined included moving window size, orientation and pixel displacement. In this study, an effort was made to understand how the accuracy of LAI estimation changed with varying orientation, displacement and window size of texture features. Four orientations (horizontal 0°, right-diagonal 45°, vertical 90°, left-diagonal 135°) and seven displacement values (3, 5, 7, 9, 11, 13 and 15 pixels) were chosen. Seven moving window sizes (3 × 3, 5 × 5, 7 × 7, 9 × 9, 11 × 11, 13 × 13 and 15 × 15) were tested with a panchromatic Quickbird image. Three texture measures that were computed from each GLCM parameter combination provided 1372 texture variables. This study can help to improve understanding of the relationship between broad-leaved forest LAI and texture features in very high spatial resolution imagery, and also provide suggestions for the selection of GLCM parameters when textural information is used to estimate forest LAI values across a large area.

## Results

### Effects of the orientation parameter on the accuracy of LAI estimation

**Figure 1:**
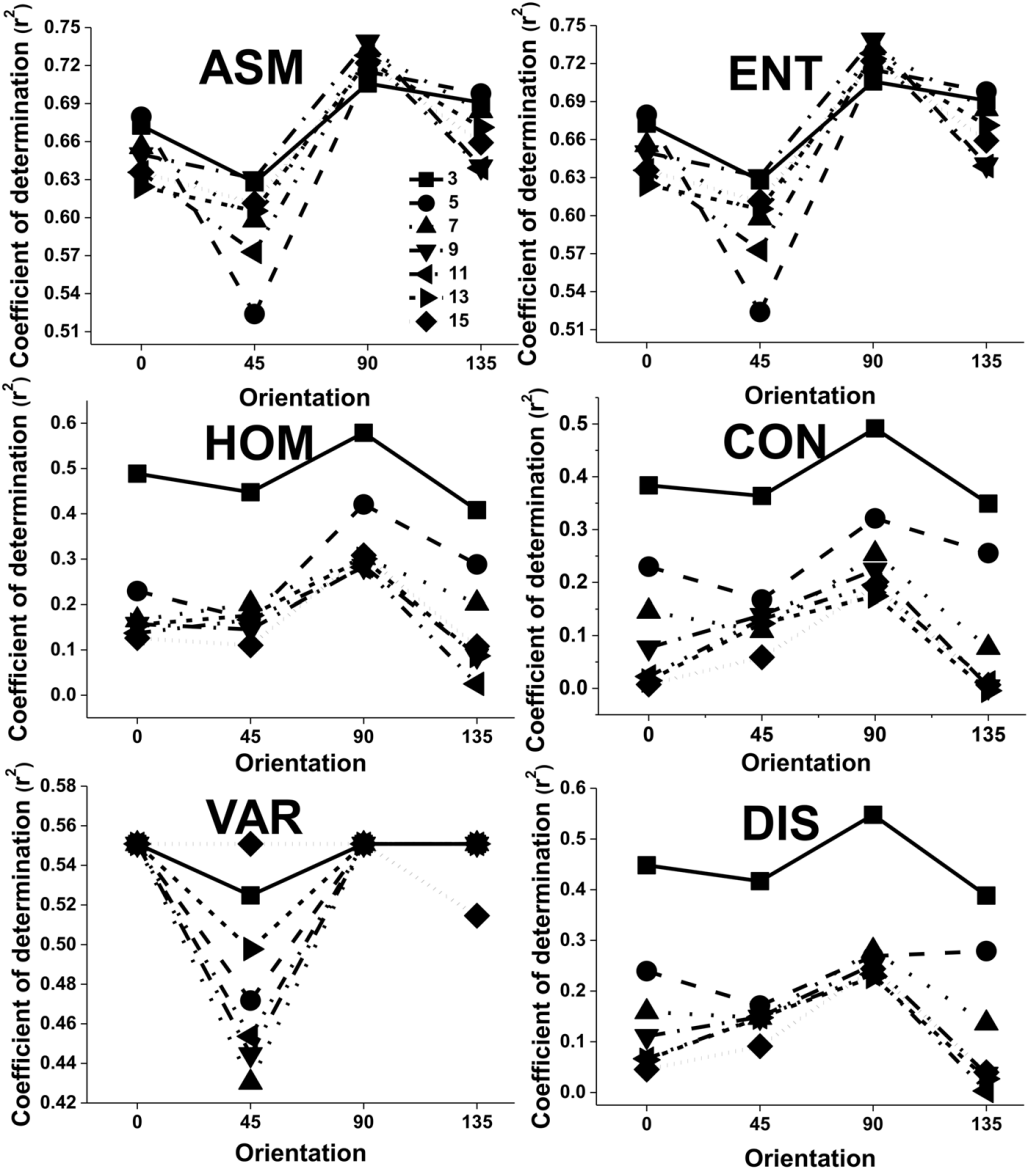
The effect of the orientation parameter on the values of adjusted r^2^ for different texture features calculated using a 3 × 3 moving window size and 3, 5, 7, 9, 11, 13 and 15 pixels (ASM, CON, COR, DIS, ENT, HOM and VAR are abbreviations for Angular Second Moment, Contrast, Correlation, Dissimilarity, Entropy, Homogeneity and Variance, respectively).

**Figure 2:**
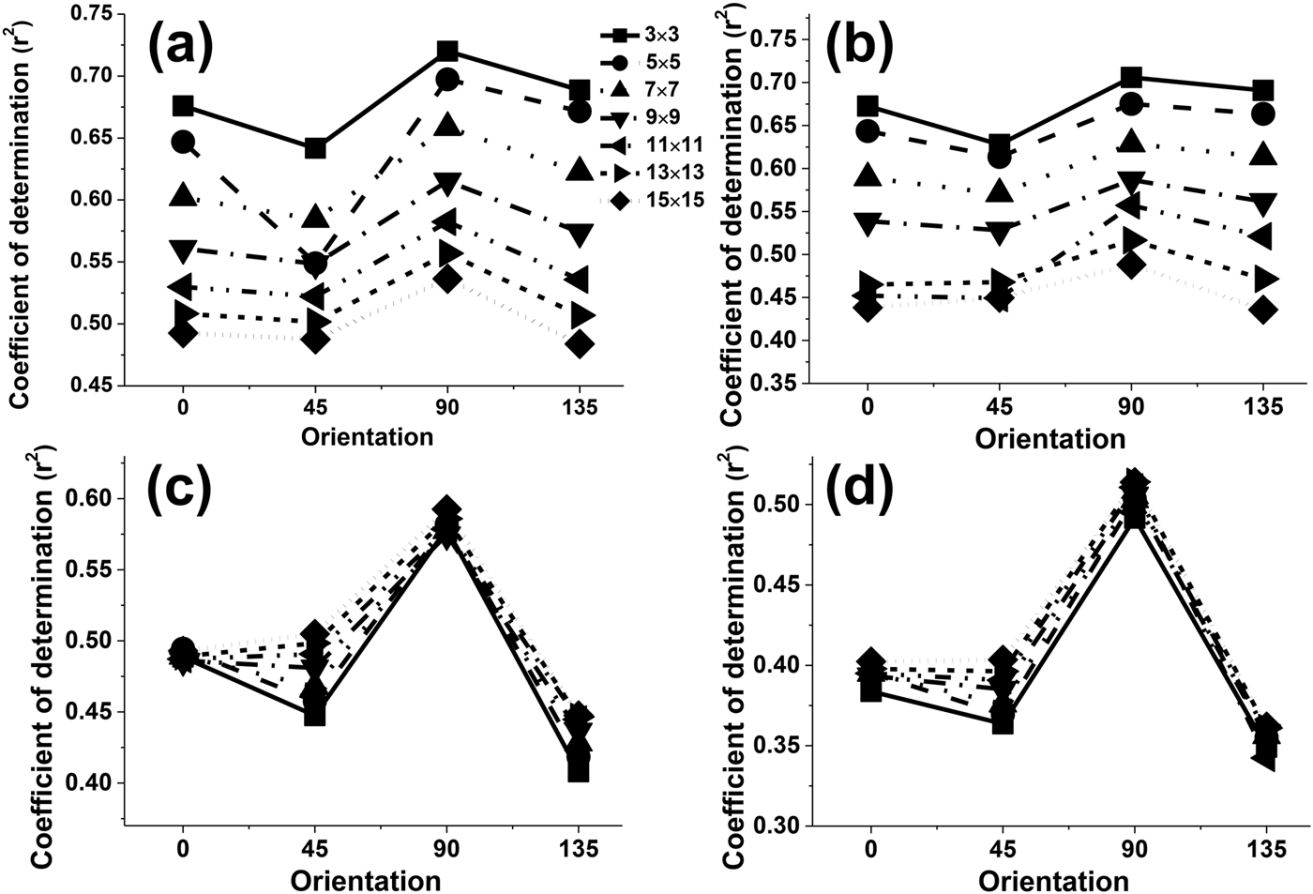
The effect of the orientation parameter on the values of adjusted r^2^ for different texture features calculated using 3 pixel displacement and 3 × 3, 5 × 5, 7 × 7, 9 × 9, 11 × 11, 13 × 13, and 15 × 15 moving window sizes.

Testing was carried out to determine an appropriate orientation for extracting texture features from a panchromatic Quickbird image. The coefficient of determination r^2^ was clearly sensitive to the orientation. The influence of orientation on the accuracy of the LAI estimation was similar for different texture features when they were calculated using a 3 × 3 moving window size and displacement values of 3 pixels (Figs 1 and 2). The lowest adjusted r^2^ value for all texture features was observed for calculations using the 45° orientation. The best choice of orientation to estimate LAI was 90° for all texture features. Using a fixed 3 × 3 window size, Fig. 1 shows the test results for six texture features and seven displacements: 3, 5, 7, 9, 11, 13 and 15. As can be seen, the 90° orientation still created a slightly better adjusted r^2^ across the six texture features. With a fixed displacement of 3 pixels, we further tested the effects of four directions (0°, 45°, 90°, and 135°) on the adjusted r^2^ with the four texture features and seven moving window sizes: 3 × 3, 5 × 5, 7 × 7, 9 × 9, 11 × 11, 13 × 13 and 15 × 15. The test results for the four texture measures with each of the four directions are shown in Fig. 2. Use of 90° orientation gave a slight improvement in the adjusted r^2^ value compared to the other directions.

### Effects of the displacement parameter on the accuracy of LAI estimation

**Figure 3:**
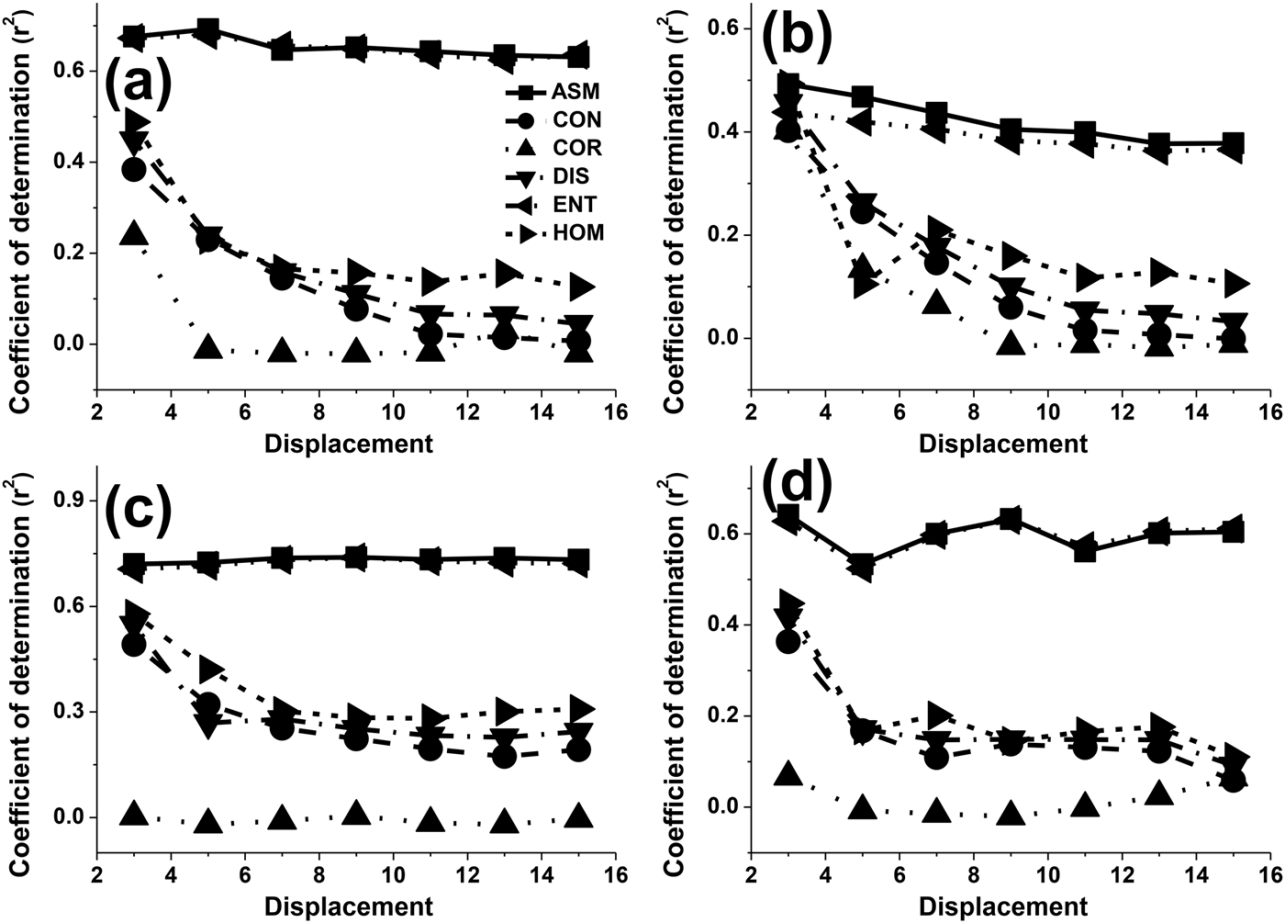
The effect of the displacement parameter on the values of adjusted r^2^ for different texture features when the orientation was set to 0° and the window size was set to 3 × 3 pixels (**a**); when the orientation was set to 0° and the window size was set to 15 × 15 pixels (**b**); when the orientation was set to 90° and the window size was set to 3 × 3 pixels (**c**); when the orientation was set to 45° and the window size was set to 3 × 3 pixels (**d**).

**Figure 4:**
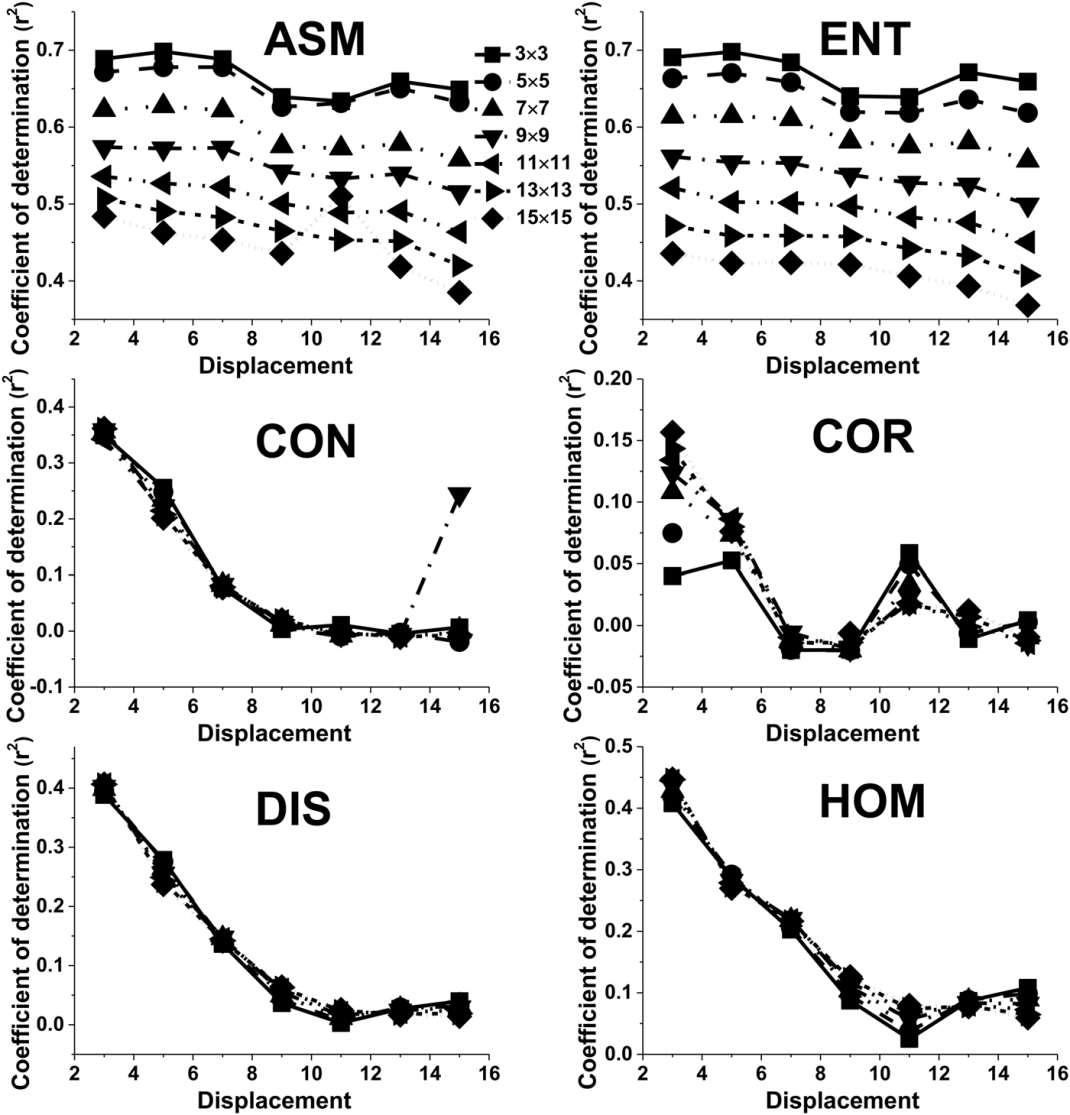
The effect of the displacement parameter on the values of adjusted r^2^ for different texture features when the orientation was set to 45° and the window size was set to 3 × 3, 5 × 5, 7 × 7, 9 × 9, 11 × 11, 13 × 13, and 15 × 15 pixels.

The adjusted r^2^ values for Contrast (CON), Correlation (COR), Dissimilarity (DIS) and Homogeneity (HOM) decreased for the most part with increasing displacement when the orientation was set to 0° and the window size was set to 3 × 3 pixels (Fig. 3a). This trend was not influenced by the window size. Compared to a window size of 15 × 15 pixels, the highest values of adjusted r^2^ were also observed for a 3 × 3 pixel window size (Fig. 3b). The orientation had a slight influence on this trend (Fig. 3a,c and d). The adjusted r^2^ values of all the texture measures as a function of the displacement showed similar trends based on the orientation and the window size when the orientation was set to 90° (Fig. 3c), 45° (Fig. 3d) and the window size was set to 3 × 3 pixels. Apart from these observations, ASM and Entropy (ENT) seemed to be less sensitive to the displacement than CON, COR, DIS or HOM (Fig. 4).

### Effects of the window size parameter on the accuracy of LAI estimation

**Figure 5:**
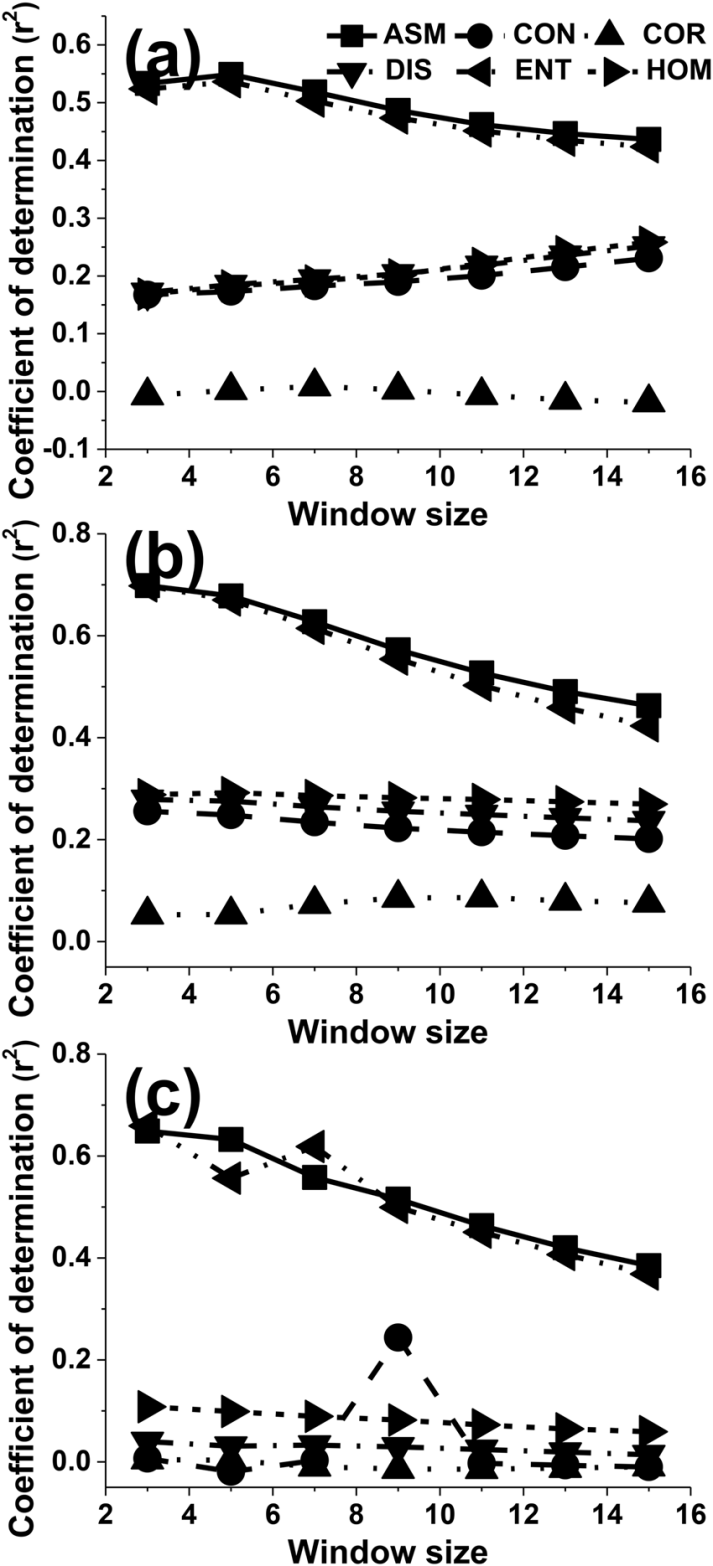
The effect of the window size on the values of adjusted r^2^ for different texture features when the orientation was set to 45° and the displacement was set to 5 pixels (**a**); when the orientation was set to 135° and the displacement was set to 5 pixels (**b**); when the orientation was set to 135° and the displacement was set to 15 pixels (**c**).

**Figure 6:**
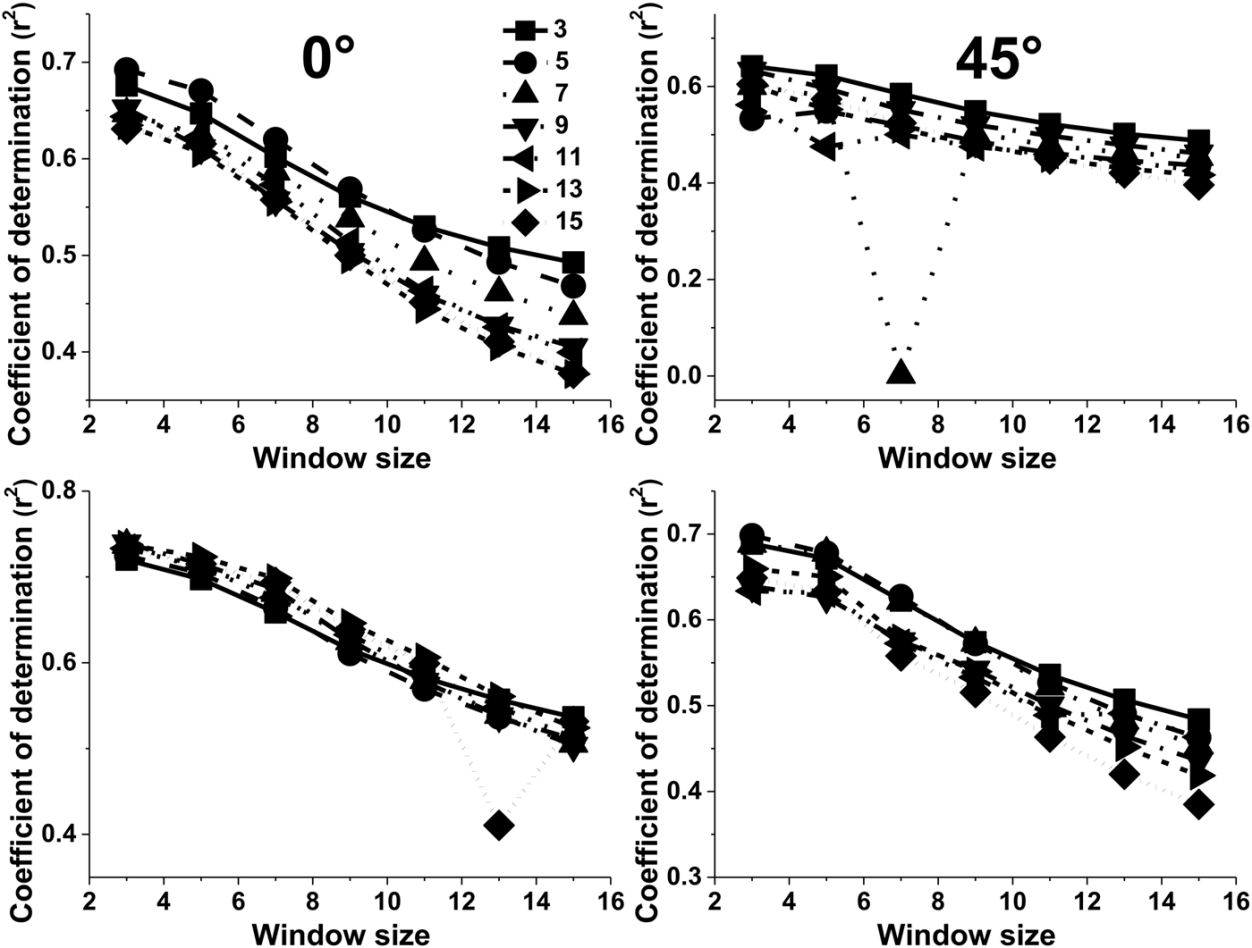
The effect of the window size on the values of adjusted r^2^ for ASM.

**Figure 7:**
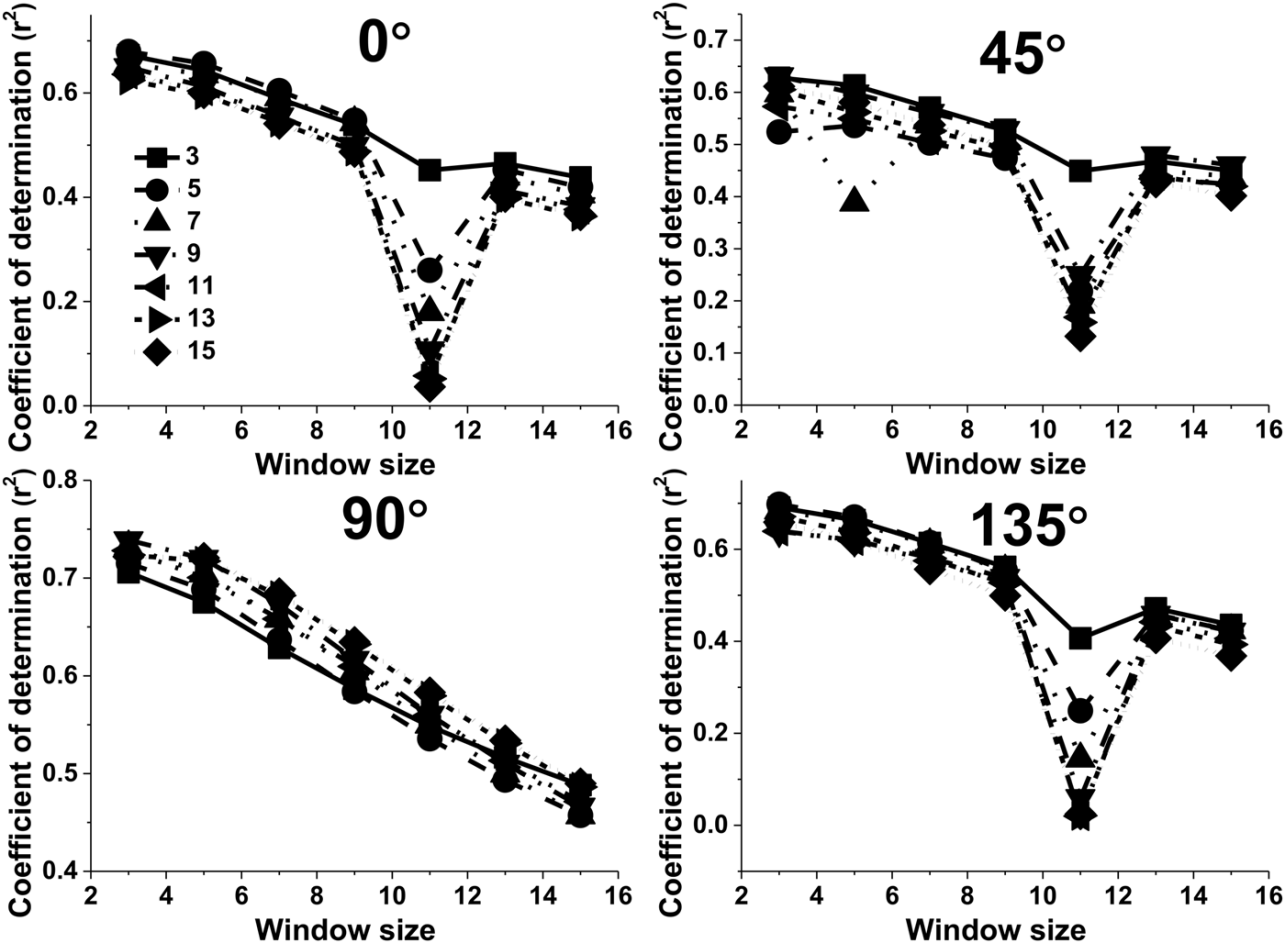
The effect of the window size on the values of adjusted r^2^ for ENT.

**Figure 8:**
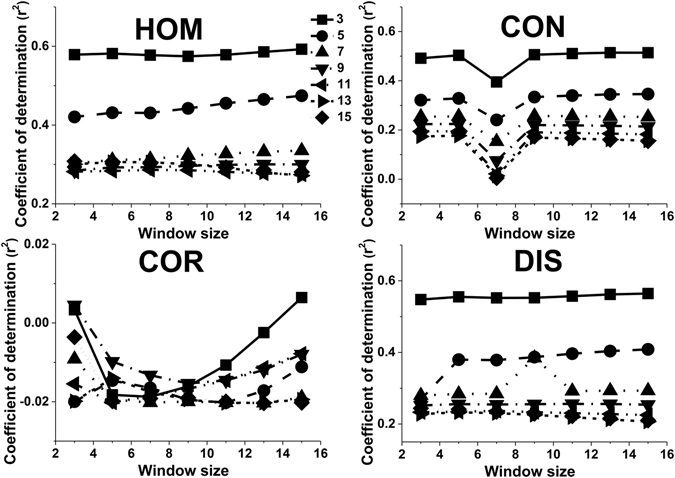
The effect of the window size on the values of adjusted r^2^ for different texture features when the orientation was set to 90°.

The moving window size was also an important factor influencing the adjusted r^2^ values. The performance of ASM and ENT decreased with increasing moving window size, with the highest adjusted r^2^ value obtained for a 3 × 3 pixel window when the orientation was set to 45° and the displacement was set to 5 pixels (Fig. 5a). For CON, COR, DIS and HOM parameters, the moving window size had little effect on the adjusted r^2^ value regardless of the values of displacement and orientation (Fig. 5a,b and c). Therefore, the displacement and orientation did not obviously affect the influence that the moving window size had on the adjusted r^2^ values. Figures 6 and 7 showed that adjusted r^2^ values obtained for ASM and ENT clearly decreased as the window size increased, regardless of the values selected for the displacement and orientation parameters. The highest adjusted r^2^ values of 0.73 and 0.74 were obtained using the 3 × 3 moving window size. On the other hand, the moving window size had nearly no effect on the retrieval results of HOM, CON and DIS when the orientation was set to 90°, regardless of the value selected for the displacement parameter. With respect to COR, adjusted r^2^ values decreased initially and then increased (Fig. 8).

## Discussion

Our study indicated that orientation, displacement and moving window size had a significant influence on the accuracy of LAI estimation when Quickbird imagery was used to calculate the GLCM parameters. Moreover, our study confirmed the results of previous work showing that the effect of orientation is greater than that of displacement^20^.

Most studies have tended to use the average value or all the values using 0°, 45°, 90° and 135° orientations to estimate forest variables and generate a forest classification^17, 21–25^. They have often neglected to investigate the orientation feature of GLCM parameters. For example, Franklin and Peddle^26^ indicated that there was a significant increase in classification accuracy when all four orientations of co-occurrence (0°, 45°, 90° and 135°) were used when compared to average textures. However, the influence of orientation on the accuracy of forest variable estimation has rarely been studied. In our study, the orientation was an important factor related to the accuracy of LAI estimation (Figs 1 and 2). The highest value of adjusted r^2^ was obtained when we used an orientation of 90° to compute the GLCM features and 45° was the worst choice when estimating the LAI value. The displacement and moving window size did not affect this trend (Figs 1 and 2). These results were the same as those reported by Pu & Cheng^9^, who demonstrated that an orientation of 90° might be used to calculate the eight second-order texture measures from all eight WV2 bands and thus obtain the highest accuracy of LAI estimation. However, our results were different to those obtained by Kayitakire *et al*.^15^, which indicated that the orientation parameter had minimal effect on the R^2^ values, especially when the displacement parameter was set to 1 pixel, even when it influenced the values of the texture features. In their study, R^2^ obtained with CON and COR was smaller in the 45° and 135° orientations than in the 0° and 90° orientations. Our results were different to those of Kayitakire *et al*.^15^ because a different image type was used and different forest variables were estimated. First, in the study by Kayitakire *et al*.^15^, texture features were extracted from digitized orthophotos with a spatial resolution of 0.80 m. Spatial resolution was close to that of IKONOS panchromatic data but different to remote sensing image data. Second, the forest variables estimated by Kayitakire *et al*.^15^ included age, top height, circumference, stand density and basal area, rather than LAI.

The accuracy of the estimate of the forest variables is greater in the 45° and 135° orientations than in the 0° and 90° orientations. According to Barber *et al*.^20^, the orientation and displacement parameters had a significant effect on the statistical distribution of textural features when they used texture parameters to determine the area of sea ice. Moreover, results obtained with an orientation of 0° were significantly better than with either 45° or 90° because 0° was parallel to the look direction of the sensor. In our study, the best results were obtained at an orientation of 90°, which was parallel to the look direction of the Quickbird imaging sensor. So, when GLCM texture parameters are used to estimate forest LAI, it appears to be better to select such an orientation.

The displacement determined the distribution of factors in the GLCM. So, it is critical to select a suitable pixel interval. Most studies set the displacement to 1 pixel when they used the GLCM method to calculate textural values. How the displacement affected textural values and LAI estimation has not been widely discussed in the recent literature^27^. In our study, the adjusted r^2^ values obtained from GLCM texture parameters nearly all decreased with increasing displacement. This might be related to the heterogeneous forest structure of the black locust plantation, where trees have a clumped distribution^26^. Larger displacements may not reflect completely the non- uniform and non-random spatial distribution of black locust plantations. The adjusted r^2^ values of CON, COR, DIS, HOM were more obviously affected than those for ASM and ENT (Figs 3 and 5a). The fact that ASM and ENT behave differently may be attributed to variations in texture dimensions. ASM and ENT describe the primitive elements that comprise an image. Other texture parameters describe spatial dependence or interactions between these texture primitives^28, 29^. Kayitakire *et al*.^15^ concluded that COR values decrease with increasing displacement, as was the case in our study. However, the change in CON was different to the result of our study and changes in other parameters as a result of changes in displacement were not observed.

Window size influences the resultant texture, possibly due to the amount of variance included^30^. Small window sizes were more sensitive to interpixel differences in the proportions of tree crown and shadow, whereas a larger window might not extract texture information efficiently due to over-smoothing of textural variations^17, 31^. In this study, CON, DIS and HOM achieved the highest accuracy of LAI estimation using 3 × 3 or 5 × 5 moving windows when the orientation was set to 135°; for ASM and ENT, the best accuracy with these window sizes occurred with a displacement of 45°. The displacement did not influence the trend in the changes of adjusted r^2^ related to moving window size. ASM and ENT were constant, regardless of displacement and orientation. These results contradict the findings of Colombo *et al*.^32^, who reported that the best textural indicator for this purpose was the dissimilarity index, computed using a 6 × 6 pixel window. Zhou *et al*.^3^ indicated that a 3 × 3 or 5 × 5 moving window size was most suitable for estimating LAI values but did not investigate the effects of displacement and orientation variation. The GLCM parameters that most influenced the estimates of forest LAI were displacement, moving window size and orientation. Our study indicated that their effects were interrelated and complex. The optimum selection of window size is dependent on the spatial resolution of the image, the spatial characteristics of the forest and sun-target-sensor geometry during image acquisition.

The reason why texture features were used to estimate LAI was to avoid the problem of SVI saturation in regression models when LAI is larger than 3. In our study, the accuracy of using texture parameter to estimate the LAI of a black locust plantation was tested. However, we did not analyze relationships between different LAI values and the parameters statistically. The main reason for this was that a large quantity of field LAI data, ranging from small to large values, would have been necessary to develop a robust model of LAI estimation using texture features measured with a large number of parameter combinations. We tried our best to choose plots with full representation and ensure a random distribution. The LAI values ranged from 0.95 to 6.80. The trees in 76 plots had differing ages, ranging from 9 years to greater than 50 years old. The plots had different slopes and aspects including shaded and sunny aspects. If we separated the field LAI data into two parts or more (LAI less than 3 and larger than 3), the correlation between field data and texture features became weak.

## Conclusions

The experimental results show the best parameter values (orientation, displacement and moving window size) to choose when calculating GLCM features from high spatial resolution imagery to estimate forest LAI values. The best orientation was 90° for estimation of LAI. A displacement of 3 pixels outperformed other displacements significantly. Using a 3 × 3 moving window size could lead to even higher accuracy of a LAI estimation than other window sizes. The high resolution of the Quickbird imagery can offer detailed textural information that is potentially helpful in estimating more accurate LAI. However, the textural information is very complex and the influences of orientation, displacement and moving window size on the accuracy of any LAI estimation are interconnected. Our study tested the sensitivity of GLCM parameters on the estimation of forest LAI. There are few studies from the existing literature that can confirm our findings, and there appear to be no studies that have examined, in detail, the influence of GLCM parameters on the evaluation of LAI. Our study only considered broad-leaved forest i.e. black locust plantations and only used the panchromatic band of imagery from the Quickbird satellite. More testing and validation work is needed, in particular using various forest ecosystems and different satellite sensors.

## Materials and Methods

### Study area

All LAI measurements were taken within the experimental area known as Huaiping forest farm which is located in Yongshou County of Shaanxi Province on the Loess Plateau of China. Its elevation ranges from 1113 to 1417 metres above sea level. The area studied was 258 km^2^. The forest vegetation is primarily composed of black locust (*Robinia pseudoacacia* L.), *Platycladus orientalis* (L.) Franco and *Pinus tabulaeformis* Carr. Black locust of different ages could be found in this area. The LAI values ranged from 0.95 to 6.80, with an average of 4.43 and a median of 4.33. The DBH values of black locust trees ranged from 5.10 to 25.5 cm with an average of 11.25 (±3.45) cm. The tree height values varied from 3.8 to 31.8 m with a mean value of 11.02 (±5.28) m. The field data showed a wide range of Above Ground Biomass (AGB), from 5.68 to 169.91 t/ha. The average value was 65.94 t/ha. There is a good correlation between LAI and biomass using the model Y = −53.27 + 81.99X − 21.06X^2^ + 1.86X^3^ (where r^2^ = 0.51, F = 150.96, P = 0.00, X is LAI and Y is biomass). The canopy of the black locust plantation on the Loess Plateau was simpler than that of a subtropical or tropical forest. The understorey layer was mainly composed of grasses with no herbs. The mean temperature was 7 °C to 13.3 °C and annual mean precipitation was 600.6 mm. The local growing season usually starts in early April and lasts until late October^33^.

### Ground-based LAI measurements

**Figure 9:**
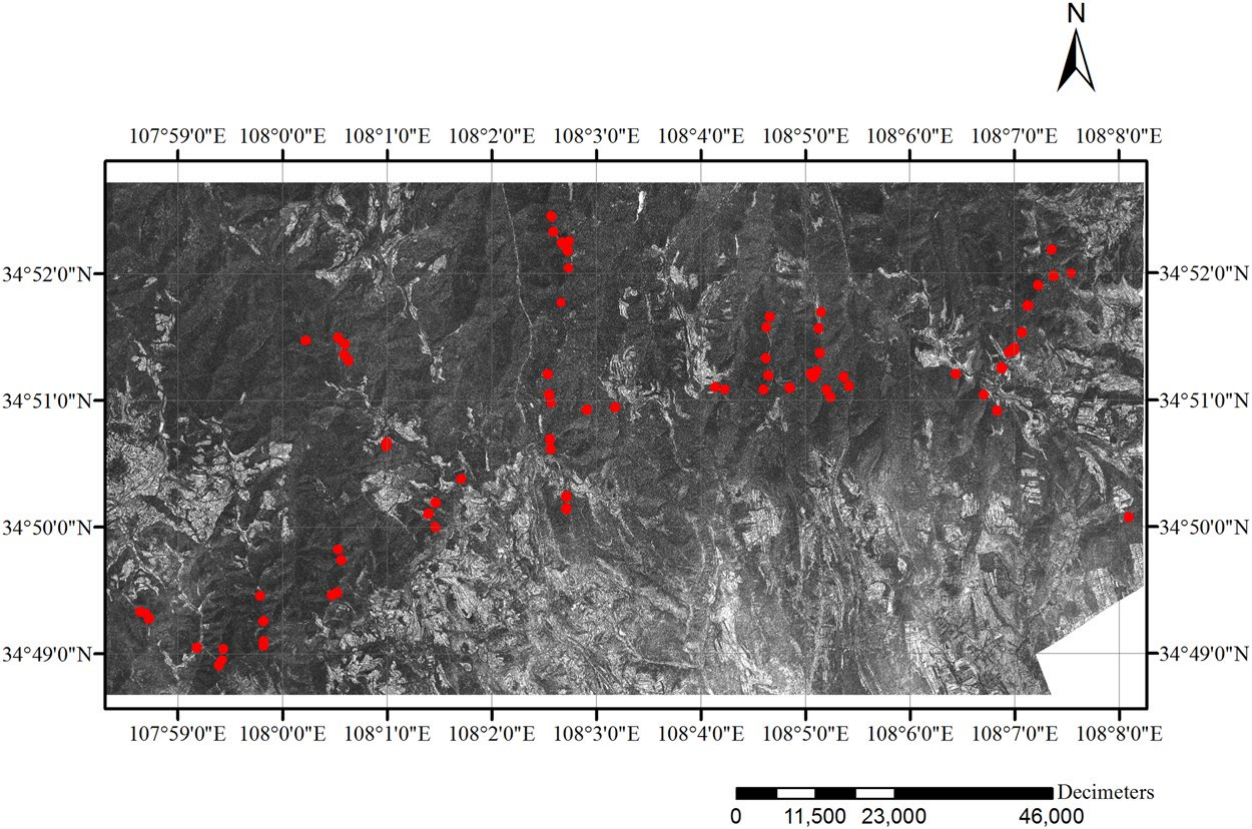
A subset of ASM features (which was calculated using a 3 × 3 moving window size, 3 pixel displacement and 135° orientation) and the location of the sample plots in the Loess Plateau region of Yongshou County, Shaanxi Province, China. The figure was created using Arcgis software package (version 10.2, http://www.esrichina.com.cn/softwareproduct/ArcGIS) for Windows.

LAI measurements were made from 16 June to 15 July 2012, under diffuse radiation conditions at sunrise and sunset, using a single sensor. The LAI-2200 instrument (LI-COR Inc., Lincoln, NE, USA; Li-Cor, 2010) was used to indirectly measure LAI in 76 black locust plantation plots. Their location is shown in Fig. 9. At each site, two above-canopy and nine below-canopy readings were taken with an opaque, 180° view-restricting cap placed over the sensor in order to mask out the operator. Setting Ring 5 was excluded from these analyses in order to obtain the most accurate LAI estimates possible.

### Remote sensing data and data pre-processing

This study was based on a single Quickbird panchromatic image that was acquired on 22 June 2012 under clear sky conditions. The panchromatic image had a spatial resolution of 0.6 m. The solar azimuth angle was 108.3° and the solar elevation angle was 66.1°. Fifty well-distributed ground control points (GCPs) and a high-resolution (1:10000) digital elevation model were used to orthorectify the data. The overall error was 0.68 pixels.

### Texture analysis

The GLCM method suggested by Haralick *et al*.^11^ was employed to measure texture features using a Quickbird panchromatic image. A GLCM is a symmetric matrix with each value representing the probability values of nearest neighbour grey tone at a given distance and orientation^28^. It reveals the spatial arrangement of grey levels in an image object. The seven features HOM, CON, DIS, ENT, VAR, ASM and COR were considered the most relevant for remote sensing analysis^14^ and were used in this study. Orientation, moving window size and displacement were the most important features influencing the values of GLCM parameters. In this study, four main orientations (0°, 45°, 90°, 135°) and seven moving window sizes (3 × 3, 5 × 5, 7 × 7, 9 × 9, 11 × 11, 13 × 13, 15 × 15) were chosen. The displacement values were set to odd numbers varying from 3 to 15. The three texture features that were computed from each GLCM parameter combination produced 1372 texture variables. Equations 1 to 7 were used for calculating the texture parameters. P(i, j) is the frequency that two pixels occur in the image, one with grey level i and the other with grey level j.

1 $${\rm{Homogeneity}}\,({\rm{HOM}})=\sum _{i,j=0}^{N-1}i\frac{{P}_{i,j}}{1+{({\rm{i}}-{\rm{j}})}^{2}}$$

2 $${\rm{Contrast}}\,({\rm{CON}})=\sum _{i,j=0}^{N-1}i{P}_{i,j}{({\rm{i}}-{\rm{j}})}^{2}$$

3 $${\rm{Dissimilarity}}\,({\rm{DIS}})=\sum _{i,j=0}^{N-1}i{P}_{i,j}|i-j|$$

4 $${\rm{Entropy}}\,(\mathrm{ENT})=\sum _{i,j=0}^{N-1}i{P}_{i,j}(-{{\rm{lnP}}}_{{\rm{i}},{\rm{j}}})$$

5 $${\rm{Variance}}\,(\mathrm{VAR})=\frac{\sum _{i,j=0}^{N-1}{({P}_{i,j}-\mu )}^{2}}{N-1}$$

6 $${\rm{Angular}}\,{\rm{Second}}\,{\rm{Moment}}\,({\rm{ASM}})=\sum _{i,j=0}^{N-1}i{{P}_{i,j}}^{2}$$

7 $${\rm{Correlation}}\,({\rm{COR}})=\sum _{i,j=0}^{N-1}ij{P}_{i,j}-{\mu }_{1}{\mu }_{2}$$

$${\mu }_{1}=\sum _{i=0}^{N-1}i\sum _{j=0}^{N-1}{P}_{i,j}$$

$${\mu }_{2}=\sum _{j=0}^{N-1}j\sum _{j=0}^{N-1}{P}_{i,j}$$

$${\sigma }_{1}^{2}=\sum _{i=0}^{N-1}{(i-{\mu }_{1})}^{2}\sum _{j=0}^{N-1}{P}_{i,j}$$

**Figure 10:**
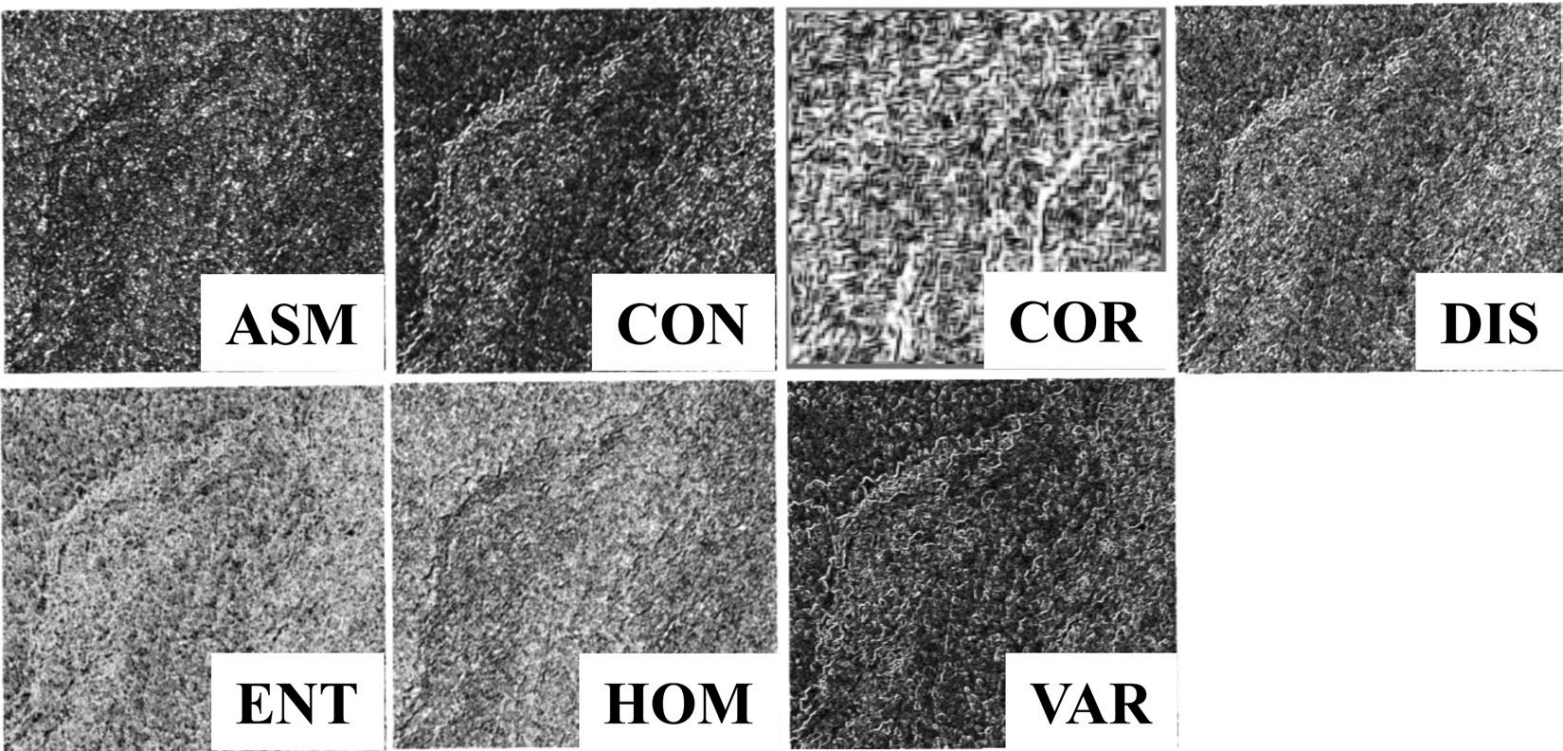
Subsets of ASM, CON, COR, DIS, ENT, HOM and VAR (which were calculated using a 3 × 3 moving window size, 3 pixel displacement and 135° orientation).

HOM is a measurement of lack of variability or the amount of local similarity in the scene. High HOM values suggest small grey tone differences in pair elements. CON is a measure of the amount of local variation in pixel values between neighbouring pixels. It is high for regions exhibiting large local variations and is the opposite of HOM. DIS is similar to CON and inversely related to HOM. It is high when the local region has a high CON. ENT is a measure of the degree of disorder in an image. ENT is larger when the image is texturally non-uniform or heterogeneous. It is the opposite of ASM. VAR is high when there is a large standard deviation of grey level in the local region. ASM and uniformity are measures of textural uniformity and pixel pair repetition, respectively. They are high when the GLCM is locally homogenous; they are similar to HOM. COR is a measure of grey level linear dependencies in the image. High COR values denote a linear relationship between the grey levels of pixel pairs. Figure 10 shows subsets of seven texture features.

### Statistical analysis

Empirical relationships between texture parameters and LAI were investigated by carrying out linear analyses using LAI as the independent variable and the texture parameter as the dependent variable. Different texture features were extracted for all field plots using an area of interest mask (AOI) of 20 m × 20 m. The average of AOI texture values was used to establish the simple linear models. Adjusted r^2^ values were computed based on linear relationships between the two variables, as required^34^. All statistical analyses were carried out using the SAS software package (version 8.0) for Windows.
